# Supplementary material for: Effectiveness of orally-delivered double-stranded RNA on gene silencing in the stinkbug Plautia stali
Source: PLoS One. 2021 Jan 14;16(1):e0245081. doi: 10.1371/journal.pone.0245081 (PMC7808618; doi:10.1371/journal.pone.0245081)
Supplement: Attachment — Submitted filename: Comments to the authors_PlosOne.docx [file pone.0245081.s003.docx]

The main objective of this study was to compare the efficiency of RNAi by injection vs feeding in the brown-winged green stinkbug *Plautia stali* (Hemiptera: Pentatomidae). Authors concluded that *P. stali* shows high sensitivity to RNAi with injected dsRNA but, unlike the allied pest stinkbugs Halyomorpha halys and Nezara viridula, very low sensitivity to RNAi with orally delivered dsRNA. Overall, this article is well presented and organized. The following suggestion might strengthen the manuscript and make it suitable for publication in PlosOne.

1. It would be important to highlight the novelty of the study. Are any of the target genes were tested before by other researches? RNAi background on *Plautia stali* is missing.

2. Authors should explain why the concentrations of dsRNA tested through injection vs feeding were different. In order to allow a direct comparison.

3. The method section is missing details. A separate section should be created for the feeding assays and injection.

4. It is not clear whether the insects were starving before exposing the insects to diets supplemented with dsRNA.

5. Authors should consider the use of cationic nanoparticles to increase the effectiveness of oral delivery.
